# An isochronic substitution benefit study of the effects of screen time on the cognitive abilities of 3–6 children

**DOI:** 10.3389/fpsyg.2024.1421341

**Published:** 2024-07-24

**Authors:** Chang Zhenya, Zhu Aifeng, Wang Ling

**Affiliations:** ^1^College of Preschool Education, Changsha Normal University, Changsha, Hunan, China; ^2^Oudun Kindergarten of Changsha, Changsha, China; ^3^College of Sports Science, Changsha Normal University, Changsha, Hunan, China

**Keywords:** screen time, cognitive ability, isochronous substitution benefit, preschoolers, language ability, math ability

## Abstract

**Purpose:**

To investigate the impact of substituting screen time with other activities on children's cognitive ability.

**Method:**

A total of 583 children (299 males and 284 females), aged 3–6 years, were selected as participants. Correlation, regression, and isochronic substitution analyses were used.

**Results:**

Screen entertainment time on TV (SET_TV) was negatively associated with children's math ability. However, screen learning time on other electronic devices besides TV (SLT_OED) and non-screen learning time by learning alone (NSLT_LA) were positively associated with math ability and language ability. After controlling for gender, age, and family socio-economic status, SET_TV remained negatively associated with math ability, while NSLT_LA remained positively correlated. Furthermore, substituting 10 min of SET_TV with NSLT_LA resulted in an increase of 0.55 in language ability and 0.87 in math ability. Similarly, substituting SLT_OED, sleeping at home, and exercising outside of kindergarten for 10 min of SET_TV resulted in an increase of 0.90, 0.43, and 0.61 in math ability, respectively.

**Conclusions:**

There are cognitive benefits when screen recreation time is replaced with screen learning time, non-screen learning time, sleep time, and exercise time, with the highest benefits observed when screen recreation time is substituted with NSLT_LA.

## 1 Introduction

Since the introduction of television in the twentieth century, people's senses of hearing and sight have been expanded to infinity. Television has brought every corner of the world together and continues to play a significant role in people's daily lives, particularly for young children and the elderly. However, with the rapid development and constant updates of electronic products such as computers, cell phones, and tablets, there is a growing trend of these devices replacing television as the primary source of entertainment and information. As people become increasingly dependent on electronic devices, their screen time also continues to increase. This has led to a steep rise in the number of people with “low head” posture and screen addiction, a phenomenon that is now spreading to preschool-aged children. Shockingly, 24% of children begin using screens before age 1, and 76% before age 2. Even among 3-year-old children who have just entered kindergarten, a staggering 78.6% spend more than the recommended daily screen time which is no more than 60 min per day (Wang et al., 2024).

Screen time, also known as screen exposure, screen-based behavior, or screen viewing behavior, is inconsistently named or translated in current research. Numerous studies have shown that excessive screen time is associated with negative effects on early childhood development, including physiological (Foreman et al., 2021; Xie and Chen, 2022; Liu et al., 2023), motor (André and Cochetel, 2022), cognitive (Li et al., 2022; Vanderloo et al., 2022b), emotional (Li and Liu, 2022; Xiang et al., 2022), and social aspects (Chang and Wang, 2022; Li and Liu, 2022; Xiang et al., 2022; Wang et al., 2024), and can even have a detrimental impact on children's physical and mental health in the long term (Ennemoser and Schneider, 2007; Vanderloo et al., 2022a). Many countries and organizations have issued guidelines related to screen time, suggesting that people should reduce screen time in early childhood (Cliff et al., 2017; Tremblay et al., 2017), and some guidelines even point out that the less screen time the better (Guan et al., 2020). For a while, screen time has been like a scourge, and parents and educators have been actively working on various strict programs to limit children's screen time. Of course, many scholars take a different view: Kostyrka-Allchorne et al. (2017) systematically reviewed the literature on the relationship between television viewing and children's executive functioning, academic performance, attention, language, and play, and showed that viewing high-quality educational content predicted preschool children's academic performance later in life. Empirical studies have also further suggested that the frequency of educational screen activities (puzzle games, online learning, etc.) moderates the negative predictive effect of screen time on children's early literacy skills (Li et al., 2022). A recent cohort study using a large population (15,965 preschoolers) showed that children's mental health risk increased significantly when screen time exposure exceeded 1 h per day, regardless of the type of screen content viewed by preschoolers, and that the risk increased over time; for the same amount of screen time viewed, children who watched entertainment and non-children's programs were at greater risk of mental health problems than those who watched educational programs. Children's mental health risk is higher when they watch entertainment and non-children's programs compared to educational programs (Wang et al., 2024).

At the same time, numerous scholars have identified key factors that significantly influence the cognitive abilities of preschool children. For example, Ma et al. (2022) conducted a comprehensive search of databases such as PubMed, Web of Science, Eric, SPORT Discus, Academic Search Premier, MEDLINE, CNKI, Wanfang, and Wipro, and analyzed 43 empirical studies to conclude that physical activity has a positive impact on the cognitive development and academic performance of young children. Similarly, Xing et al. (2018) found that longer sleep duration is associated with better brain development and executive function in toddlers and children, with nighttime sleep ratio being a significant predictor of executive function after 3 months. Executive function is considered to be the most advanced process of human cognitive activity, encompassing a wide range of cognitive functions that develop rapidly in early childhood (Luan et al., 2013). In fact, it is widely recognized as the highest level of cognitive activity in humans during this stage of development. Moreover, recent guidelines for preschoolers' daily activities and related studies have revealed a strong connection between physical activity, sleep, and screen time. It is important to note that the total amount of time spent on physical activity, sleep, and sedentary behavior, including screen time, make up a 24-h day. This highlights the need for researchers to adopt a holistic perspective when studying the effects of screen time (Chang et al., 2020a). In other words, researchers must consider the overall balance of a child's daily activities when examining the impact of screen time.

In summary, while there has been extensive research on the relationship between screen time and preschool children's cognitive ability, the focus has been primarily on the associations between physical activity, screen time, and sleep, with fewer studies examining these factors together. Existing studies have also primarily looked at the different effects of various types of screen activities on cognitive ability, but there is a lack of specific strategies for how to allocate screen time in a balanced way (Chang and Wang, 2022). It is often suggested that screen time for learning should be increased while screen time for entertainment should be decreased, but this overlooks the fact that screen time is just one aspect of a child's daily life and can be adjusted to other activities such as sleep, exercise, and study time. Parents should be provided with information on how to adjust screen time rules and find alternative activities for their preschoolers (De Decker et al., 2012). However, previous statistical tools have limited scholars from considering these variables holistically and have also caused issues with covariance (Chang and Wang, 2022). With the development of new statistical tools, this problem can now be addressed (Chang et al., 2020a). This study aims to explore the cognitive benefits of different types of screen time substitutions and how adjusting screen time to other parts of a child's day, such as sleep, exercise, or study time, can impact cognitive ability. This is referred to as isochronous substitution benefits in this study. Research hypothesis: (1) There is a negative correlation between screen time and cognitive abilities in children aged 3–6 years old. However, there is a positive correlation between learning time and exercise time and cognitive abilities in this age group. (2) Replacing screen entertainment time with screen learning time can have a positive impact on cognitive abilities. (3) Replacing screen time with non-screen learning activities, particularly screen entertainment time, can have a positive effect on cognitive abilities. (4) Replacing screen time with sleep and exercise, especially screen entertainment time, can have a positive impact on cognitive abilities.

## 2 Materials and methods

### 2.1 Participants

Based on Tsinghua University's survey program on the developmental status of children aged 3–6, the 2020 “Early Childhood Development Data System Collection” project was carried out in seven kindergartens in Changsha, SanYu Group. A total of 900 children were randomly selected from each of the kindergarten's three classes (junior, middle, and senior), with 702 children participating in the study due to practical constraints. After the questionnaire stage, 630 valid data were obtained, and after the cognitive ability test, 650 valid data were obtained, resulting in a total of 583 participants (299 males and 284 females; see Table 1). This study obtained ethical approval from the Ethics Review Committee (HR342-2024).

**Table 1:** Basic information about the study subjects.

		**Grade**			**Total**
		**Junior class**	**Middle class**	**Senior class**	
Sex	Male	75	129	95	299
	Female	101	97	86	284
Total		176	226	181	583

### 2.2 Materials

#### 2.2.1 Questionnaire on screen time and related factors for preschoolers

The questionnaire focuses on investigating the screen time, exercise time, sleep time, and study time of preschool children. It is completed by the primary caregiver of the child and is based on the “Home Scale” from the China Urbanization and Children Development Survey (CUCDS) at Tsinghua University. The Home Scale has been modified to suit the characteristics of preschool children. The primary caregiver is asked to report the average daily time spent on various activities in the past month, recording the hours and minutes. If the time is a whole number of hours, the minutes should be 0. If there is no such activity, please also fill in 0. The questionnaire includes the following components: ① Screen Entertainment Time (SET), which includes using the TV for entertainment (SET_TV) and using other electronic devices besides TV, such as mobile phones and tablets, etc., for entertainment (SET_OED); ② Screen Learning Time (SLT), which includes using the TV for learning (SLT_TV) and using other electronic devices besides TV, such as mobile phones and tablets, etc., for learning (SLT_OED); ③ Non-Screen Learning Time (NSLT), which includes learning alone without the use of electronic devices, such as doing homework, reading, studying, drawing, and playing the piano (NSLT_LA) and learning with parents, such as reading bedtime stories (NSLT_LWP); ④ Sleep Time at Home (STH), which includes both nighttime and daytime sleep; and ⑤ Exercise Time Outside Kindergarten (ETOK), which refers to physical activity to improve physical health. Generally, exercise should last for at least 20 min and result in shortness of breath and sweating. The total screen time (TST) is calculated by adding SET and SLT, and the total learning time (TLT) is calculated by adding SLT and NSL. Each component has two subcomponents for weekdays and weekends, and the final assessment time is calculated as “(Weekday Time^*^5+Weekend Time^*^2)/7.”

#### 2.2.2 Cognitive ability test for 3–6-year-old

The cognitive ability test in this study, also known as the learning ability test or academic ability test, was derived from the China Urbanization and Children Development Survey (CUCDS) of Tsinghua University, which was designed by Professor Houcan Zhang of Beijing Normal University and applies to Chinese children between the ages of 3 and 12 years old (Yan, 2017; Shen, 2019; Zhu and Liu, 2019). In this study, the Language Ability Test (LAT) and the Mathematics Ability Test (MAT) were utilized to evaluate the cognitive ability of children aged 3–6 years old. While cognitive ability encompasses more than just language and math skills, these two areas have commonly been used in previous studies as indicators of children's overall cognitive development (Yan, 2017; Shen, 2019).

#### 2.2.3 Questionnaire on family's socio-economic status

The SES questionnaire was developed mainly regarding Yuan et al. (2009)'s SES questionnaire from the Institute of Developmental Psychology, Beijing Normal University. The study examined the impact of parents' literacy, occupation, and economic income on adolescents. To gather information on family income, the researchers used a questionnaire that was designed to account for the fact that adolescents may not have accurate knowledge of their family's income. However, since the questionnaire was filled out by parents in this study, the indicator of family economic income was directly chosen. The researchers then used the Program for International Student Assessment (PISA) method to calculate the SES of the families (OECD, 2003). This involved four steps and the SES was divided into three levels based on a standard deviation of 1. The occupational classification was determined using the scoring criteria from the International Classification of Occupations and the Socio-Economic Status Index (ISEI) developed by Ganzeboom and Treiman (1996).

### 2.3 Process

The first stage is pre-testing. A sample of 30 children and 30 parents were selected to participate in the cognitive ability test and questionnaire completion, based on which adjustments were made to form a formal program of action for the study, while informed consent was obtained from the parents of all the children participating in the study.

In the second stage, formal testing was conducted. First, an online questionnaire was used to survey preschool children's screen time and related factors, as well as their family's socio-economic status. Due to the large number of items assessed by parents in this study, a full tracking and control measurement was used for quality control. Parents were informed of the importance of accurately assessing their child's screen time during a centralized meeting before the test. This emphasized the positive impact it could have on later home-based co-education strategies. During the test, monitoring was carried out through secondary information. Parents were provided with tips and methods for accurately assessing their child's screen time and were reminded to review their completed assessment to minimize errors. Any assessment data that seemed unreasonable, such as zero sleep time, were excluded from the analysis. Doubtful data were also verified with the parents of the subjects in question. Next, the Cognitive Ability Test for 3–6-year-olds was administered. The test was conducted individually in a quiet, well-lit room. The child (subject) was given 20 min to complete the test, with 10 min each for the language and math portions. The question-and-answer session had to be stopped immediately after the allotted time. Finally, five parents were randomly selected from the junior class, middle class, and senior class to participate in the interview. The questions were designed based on the survey results, particularly any findings that were inconsistent with previous studies. Some examples of questions asked were: (1) Which do you think is more important, independent learning or accompanied learning? Why? (2) Which do you value more, learning through screens or non-screen methods? How do you balance your child's time between these two types of learning? (3) How much screen time do you think is appropriate for your child, and how do you see different types of screen time impacting your child? Interviews were also conducted with kindergarten directors, teachers, and parents to ensure an accurate interpretation of the findings.

### 2.4 Statistics

First, to determine the normality of continuous variables, histograms, skewness, and kurtosis were considered comprehensively. Basic descriptive statistics were then performed on the collected data. Secondly, the independent samples *t*-test was used to analyze differences between the two groups, while one-way ANOVA was used for differences between more than two groups. Correlation and regression analyses were also conducted to explore the relationship between screen time, related factors, and cognitive ability (Chang et al., 2020b). Finally, isochronous substitution analysis, a popular method in the fields of public health and physical education, was used to examine the impact of substituting different types of screen time and other activities on preschoolers' cognition. This method utilizes a multiple linear regression model that includes all time-use components of interest, with one component removed at a time and used as an explanatory variable. The unstandardized regression coefficients of the included components are then interpreted as the change in the outcome variable due to a change in time spent on the excluded components (Mekary et al., 2009). Specifically, four models were constructed for the isochronous substitution analysis in this paper.

Model 1:

Y1 = (b2) SET_OED + (b3) SLT_TV + (b4) SLT_OED + (b5) NSLT_LA + (b6) NSLT_LWP + (b7) STH + (b8) ETOK + (b9) TOTAL TIME + (b10) SES + (b11) AGE.

Model 2:

Y2 = (b1) SET_TV + (b3) SLT_TV + (b4) SLT_OED + (b5) NSLT_LA + (b6) NSLT_LWP + (b7) STH + (b8) ETOK + (b9) TOTAL TIME + (b10) SES + (b11) AGE.

Model 3:

Y3 = (b1) SET_TV + (b2) SET_OED + (b4) SLT_OED + (b5) NSLT_LA + (b6) NSLT_LWP + (b7) STH + (b8) ETOK + (b9) TOTAL TIME + (b10) SES + (b11) AGE.

Model 4:

Y4 = (b1) SET_TV + (b2) SET_OED + (b3) SLT_TV + (b5) NSLT_LA + (b6) NSLT_LWP + (b7) STH + (b8) ETOK + (b9) TOTAL TIME + (b10) SES + (b11) AGE.

## 3 Results

### 3.1 Basic information on screen time and related factors for preschoolers

TLT was significantly smaller for boys than girls (*t* = −2.45, *p* = 0.015), and NSLT was significantly smaller for boys than girls (*t* = −2.27, *p* = 0.023) but there was no gender difference in both SLT (*t* = −0.52, *p* = 0.601) and TST (*t* = 0.96, *p* = 0.336) were not gender-specific; girls spent more time on studying than boys, which is generally consistent with previous studies (Jiang et al., 2014), girls often have more patience and sit still than boys in online classes, watching learning videos, and playing puzzle games, which is reflected in their higher sedentary time compared to boys (Chang et al., 2020b).

The amount of time spent on screens was found to be significantly higher for children from low and middle socioeconomic status (SES) families compared to those from high SES families, *F* = 4.14, *p* = 0.016). Similarly, SET was significantly higher for low and middle SES children than high SES children (*F* = 3.81, *p* = 0.023). On the other hand, NSLT was significantly higher for low SES children compared to high SES children (*F* = 3.27, *p* = 0.039), and STH was significantly lower for low SES children compared to middle and high SES children (*F* = 3.69, *p* = 0.026). Families with low SES children were characterized by a pattern of “double highs and one low,” with high screen time and study time, but low sleep time.

Children in the learning time of “ < 4-year-old group” and “4-year-old group” are both less than the “>4-year-old group (M_ < 4_ =107.60 ± 58.73, M_4_ =109.66 ± 55.72, M_>4_ =125.16 ± 58.05, *F* = 5.47, *p* = 0.004),” but there was no age difference in screen time (*F* = 0.77, *p* = 0.464); Sleep time at home in the “ < 4-year-old group” and “4-year-old group” of preschool children are both greater than the “>4-year-old group” (*F* = 11.97, *p* < 0.001). As young children progress through the grades, especially into senior class, parents may plan more for their children's school time to prepare for elementary school enrolment (see Table 2).

**Table 2:** Basic information on screen time and related factors in preschool children.

		***N***	**SET**	**SLT**	**NSLT**	**STH**	**ETOK**
Sex	Male (x)	299	62.09 ± 46.17	37.83 ± 31.07	67.57 ± 38.42^x < y^	570.74 ± 45.12	72.43 ± 41.69
	Female (y)	284	55.89 ± 40.31	39.21 ± 32.51	72.89 ± 36.23	577.09 ± 41.48	68.61 ± 38.33
Age	< 4 (1)	170	57.28 ± 40.51	36.00 ± 32.81	66.39 ± 36.62	582.23 ± 43.01^1>3^	70.92 ± 39.87
	4 (2)	210	62.80 ± 48.24	37.59 ± 30.80	69.21 ± 35.54	578.28 ± 41.83^2>3^	71.50 ± 42.00
	>4 (3)	203	56.72 ± 40.57	41.88 ± 31.69	74.30 ± 39.73	562.20 ± 43.25	69.32 ± 38.53
SES	Low (a)	69	65.31 ± 38.14^a>c^	39.41 ± 34.64	77.33 ± 52.85^a>c^	561.40 ± 37.76^a < b, c^	73.04 ± 43.17
	Medium (b)	420	60.44 ± 43.91^b>c^	39.54 ± 32.13	70.69 ± 35.12	574.64 ± 44.57	70.13 ± 39.55
	High (c)	94	48.35 ± 43.95	33.24 ± 27.37	62.55 ± 32.77	579.35 ± 41.03	70.71 ± 40.55
Total		583	59.07 ± 43.49	38.51 ± 31.76	70.16 ± 37.44	573.83 ± 43.47	70.57 ± 40.10

SET is Screen Entertainment Time, SLT is Screen Learning Time, NSLT is Non-Screen Learning Time, STH is Sleeping Time at Home, and ETOK is Exercise Time Outside of Kindergarten; SES is the socio-economic status of the family, SES = (0.707^*^Z literacy level + 0.747^*^Z occupation + 0.717^*^Z family income)/1.572; ^*^ represents the difference between genders, a, b, and c represent low, medium, and high levels of SES in that order, and < or > represents the direction of significant difference.

### 3.2 Basic cognitive skills of preschool children

The study found that boys had better math ability (*t* = 2.95, *p* = 0.003) than girls. Additionally, both language ability (*F* = 62.45, *p* < 0.001) and math ability (*F* = 154.21, *p* < 0.001) were lower in the “ < 4-year-old group” compared to the “4-year-old group.” Children in the “4-year-old group” also had higher cognitive ability than those in the “>4-year-old group.” These findings support the idea that cognitive ability increases rapidly with age.

Different SES groups had no significant difference in language ability (*F* = 0.36, *p* = 696) or math ability (*F* = 1.20, *p* = 0.301). While previous studies have shown that SES can impact cognitive ability, this study did not find a significant relationship between SES and language or math ability. This suggests that cognitive ability is influenced by multiple factors (see Table 3).

**Table 3:** Basic cognitive skills of preschool children.

		***N***	**Language ability**	**Math ability**
Sex	Male (x)	299	99.89 ± 13.69	100.77 ± 14.95^x>y^
	Female (y)	284	97.51 ± 15.60	97.13 ± 14.80
Age	< 4 years (1)	170	90.50 ± 11.94^1 < 2 < 3^	89.09 ± 9.95^1 < 2 < 3^
	4 years old (2)	210	98.37 ± 13.01	95.93 ± 11.52
	>4 years old (3)	203	106.00 ± 14.73	110.46 ± 14.21
SES	Low (a)	69	98.44 ± 15.60	96.99 ± 15.76
	Medium (b)	420	98.51 ± 14.65	98.95 ± 14.93
	High (c)	94	99.91 ± 14.25	101.28 ± 14.32
Total		583	98.73 ± 14.69	98.99 ± 14.98

SES stands for family socioeconomic status.

### 3.3 Correlations of screen time and related factors with cognitive ability in preschoolers

Language ability was significantly and positively correlated with both SLT_OED (*r* = 0.099, *p* < 0.05) and NSLT_LA (*r* = 0.126, *p* < 0.01), while math ability was also significantly and positively correlated with SLT_OED (*r* = 0.105, *p* < 0.05), NSLT_LA (*r* = 0.146, *p* < 0.001), math ability was significantly negatively correlated with SET_TV (*r* = −0.108, *p* < 0.01; see Table 4).

**Table 4:** Correlation of screen time and related factors with cognitive ability in preschool children.

	**ChZ**	**MaZ**	**SET_TV**	**SET_OED**	**SLT_TV**	**SLT_OED**	**NSLT_LA**	**NSLT_LWP**	**STH**
ChZ	–								
MaZ	0.690^***^	–							
SET_TV	−0.057	−0.108^**^	–						
SET_OED	0.019	0.015	0.158^***^	–					
SLT_TV	0.057	0.036	0.218^***^	0.209^***^	–				
SLT_OED	0.099^*^	0.126^**^	0.029	0.360^***^	0.329^***^	–			
NSLT_LA	0.105^*^	0.146^***^	−0.019	0.058	0.074	0.126^**^	–		
NSLT_LWP	−0.012	−0.026	−0.040	0.134^**^	0.168^***^	0.169^***^	0.210^***^	–	
STH	−0.071	−0.105^*^	−0.138^**^	−0.091^*^	−0.117^**^	−0.139^**^	−0.107^**^	−0.003	–
ETOK	0.002	0.040	0.000	−0.097^*^	0.072	−0.046	0.096^*^	0.148^***^	−0.052

SET_TV is screen entertainment time, which includes watching cartoons and other entertainment programs on TV, SET_OED is screen entertainment time which includes using other electronic devices besides TV for entertainment, SLT_TV is screen learning time which includes watching educational programs on TV, SLT_OED is screen learning time which includes using other electronic devices besides TV for learning, NSLT_LA is learning alone without the use of electronic devices, NSLT_LWP is learning with parents without the use of electronic devices, STH is sleep time at home, ETOK is exercise time outside of kindergarten, ChZ stands for language ability, MaZ stands for math ability.

^*^Stands for p < 0.05.

^**^Stands for p < 0.01.

^***^Stands for p < 0.001.

### 3.4 Effects of screen time and related factors on cognitive ability with control variables

After controlling for gender, age, and family socioeconomic status, SET_TV was negatively associated with math ability (B = −0.04, *p* = 0.004) and positively associated with NSLT_LA (*B* = −0.05, *p* = 0.011; see Table 5).

**Table 5:** Effect of screen time and related factors on cognitive ability with control variables.

**Variant**	**Regression coefficient**	**ChZ**	**MaZ**
SET_TV	B (95% CI)	−0.02 (−0.05, 0.01)	**−0.04 (−0.07**, **−0.01)**
	P	0.188	0.004
SET_OED	B (95% CI)	0.01 (−0.05, 0.07)	0.01(−0.05, 0.06)
	P	0.761	0.871
SLT_TV	B (95% CI)	0.03 (−0.02, 0.09)	0.02 (−0.03, 0.07)
	P	0.204	0.464
SLT_OED	B (95% CI)	0.05 (−0.02, 0.10)	0.05 (−0.003, 0.10)
	P	0.162	0.063
NSLT_LA	B (95% CI)	0.03 (−0.01, 0.07)	**0.05 (0.01, 0.08)**
	P	0.114	0.011
NSLT_LWP	B (95% CI)	−0.001 (−0.06, 0.06)	−0.01 (−0.06, 0.04)
	P	0.978	0.779
STH	B (95% CI)	0.0004 (−0.03, 0.03)	−0.003 (−0.03, 0.02)
	P	0.973	0.831
ETOK	B (95% CI)	0.003 (−0.02, 0.03)	0.02 (−0.01, 0.04)
	P	0.814	0.152

SET_TV is screen entertainment time, which includes watching cartoons and other entertainment programs on TV, SET_OED is screen entertainment time which includes using other electronic devices besides TV for entertainment, SLT_TV is screen learning time which includes watching educational programs on TV, SLT_OED is screen learning time which includes using other electronic devices besides TV for learning, NSLT_LA is learning alone without the use of electronic devices, NSLT_LWP is learning with parents without the use of electronic devices, STH is sleep time at home, ETOK is exercise time outside of kindergarten, ChZ stands for language ability, MaZ stands for math ability; Bolding indicates that the regression is significant.

### 3.5 Effects on cognitive ability after substituting different activity times for each other

After controlling for the effects of variables such as gender, age, and SES, language ability increased by 0.55 after NSLT_LA substituted for SET_TV 10 min/d; math ability increased by 0.90, 0.87, 0.43, and 0.61 after SLT_OED, NSLT_LA, sleep time at home, and exercise time outside kindergarten respectively substituted for SET_TV 10 min/d. Additionally, when SLT_OEP and NSLT_LA replaced NSLT_LWP 10 min/d, math ability increased by 0.88 and 0.85, respectively (see Table 6).

**Table 6:** Changes in preschool children's cognitive abilities after screen time and related factor time were substituted for each other for 10 min/d.

	**SLT_TV**		**SLT_OED**		**NSLT_LA**		**NSLT_LWP**		**STH**		**ETOK**	
	**B**	***P***	**B**	***P***	**B**	***P***	**B**	***P***	**B**	***P***	**B**	***P***
	**(95% CI)**		**(95% CI)**		**(95% CI)**		**(95% CI)**		**(95% CI)**		**(95% CI)**	
Δ**ChZ**												
Model 1 (replaces SET_TV)	0.61	0.096	0.55	0.128	**0.55**	0.033	0.01	0.988	0.27	0.157	0.26	0.213
	(−0.11, 1.33)		(−0.16, 1.26)		(0.05, 1.05)		(−0.64, 0.65)		(−0.11, 0.65)		(−0.15, 0.68)	
Model 2 (alternative to SET_OED)	0.38	0.410	0.32	0.553	0.32	0.417	−0.22	0.636	0.05	0.896	0.04	0.917
	(−0.53, 1.30)		(−0.75, 1.39)		(−0.46, 1.10)		(−1.14, 0.70)		(−0.66, 0.75)		(−0.65, 0.72)	
Model 3 (replaces SLT_TV)	–	–	−0.06	0.906	−0.06	0.864	−0.61	0.176	−0.34	0.296	−0.35	0.310
			(−1.06, 0.94)		(−0.77, 0.65)		(−1.48, 0.27)		(−0.97, 0.29)		(−1.02, 0.32)	
Model 4 (replaces SLT_OED)	0.06	0.906	–	–	−0.002	0.995	−0.55	0.249	−0.28	0.434	−0.29	0.424
	(−0.94, 1.06)				(−0.80, 0.80)		(−1.47, 0.38)		(−0.97, 0.42)		(−0.99, 0.42)	
Δ**MaZ**												
Model 1 (replaces SET_TV)	0.65	0.050	**0.90**	0.006	**0.87**	< 0.001	0.02	0.933	**0.43**	0.014	**0.61**	0.001
	(−0.0008, 1.29)		(0.26, 1.54)		(0.42, 1.33)		(−0.55, 0.60)		(0.09, 0.77)		(0.24, 0.99)	
Model 2 (alternative to SET_OED)	0.20	0.638	0.45	0.928	0.43	0.232	−0.42	0.316	−0.02	0.957	0.16	0.600
	(−0.62, 1.02)		(−0.51, 1.42)		(−0.27, 1.12)		(−1.25, 0.41)		(−0.65, 0.62)		(−0.45, 0.78)	
Model 3 (replaces SLT_TV)	–	–	0.26	0.574	0.23	0.483	−0.62	0.123	−0.21	0.459	−0.03	0.918
			(−0.64, 1.16)		(−0.41, 0.87)		(−1.41, 0.17)		(−0.78, 0.35)		(−0.63, 0.57)	
Model 4 (replaces SLT_OED)	−0.26	0.574	–	–	−0.03	0.937	**−0.88**	0.039	−0.47	0.138	−0.29	0.371
	(−1.16, 0.64)				(−0.75, 0.69)		(−1.71, −0.04)		(−1.09, 0.15)		(−0.92, 0.34)	

Row variables are substituted variables and column variables are substitutes. Bold represents significant changes after substitution. SET_TV is screen entertainment time, which includes watching cartoons and other entertainment programs on TV, SET_OED is screen entertainment time which includes using other electronic devices besides TV for entertainment, SLT_TV is screen learning time which includes watching educational programs on TV, SLT_OED is screen learning time which includes using other electronic devices besides TV for learning, NSLT_LA is learning alone without the use of electronic devices, NSLT_LWP is learning with parents without the use of electronic devices, STH is sleep time at home, ETOK is exercise time outside of kindergarten, ChZ stands for language ability, MaZ stands for math ability.

For boys, language ability increased sequentially by 1.03 and 0.51, respectively after replacing SET_TV with SLT_TV and sleep time at home for 10 min/d, and by 0.90 after replacing exercise time outside kindergarten with SLT_TV for 10 min/d; and after replacing SET_TV with SLT_TV, SLT_OED, NSLT_LA, sleep time at home, and exercise time outside kindergarten for 10 min/d math ability rose 0.96, 1.23, 0.86, 0.61, and 0.70, respectively. For girls, NSLT_LA substituted for SET_TV for 10 min/d to increase math ability by 0.75 sequentially (see Supplementary material 1).

## 4 Discussion

The present study utilized traditional multiple regression analysis and isochronous alternative analysis to examine the relationship between screen time and cognitive ability. Consistent with previous research, the results support the negative impact of screen time for entertainment, particularly television (SET_TV), on the cognitive ability of 3–6-year-olds. Furthermore, the association between screen time and cognitive ability was found to be influenced by the type of screen time used. When screen time was used for learning, the association with cognitive ability was weakened or even partially positive. This study also revealed several key findings.

First, the negative impact of TV use on children is relatively higher than the use of other electronic devices. Academic research on children's screen time began in the 1980's, and by the beginning of the twenty-first century, the composite concept of “screen time” emerged. This concept includes the use of computers, game consoles, and other electronic products, and has gradually replaced “television exposure” as a hot research topic in the field of children's health (Xie and Chen, 2022). The present study follows the historical development of TV screen time and other electronic product screen time. Through this examination, it was found that TV screen time has a significantly negative impact on children. This may be attributed to the fact that the use of other electronic products not only involves screen time but also includes time spent playing electronic games. The associations between different types of electronic games and cognitive development may vary, particularly in the case of puzzles. It is important to note that the association between different types of video games and children's cognitive development may also differ, especially in the case of educational video games, which may have a positive impact on children's cognitive abilities (Li and Liu, 2022). Educational video games may be positively associated with children's cognitive abilities. Some scholars have further revealed that action video games can promote the development of individual cognitive ability, educational games can improve learning motivation and academic performance, and pro-social video games can reduce individual aggression and improve individual pro-social behavior (Niu et al., 2014).

Secondly, SET can have cognitive benefits when it is converted to SLT, NSLT, STH, and ETOK. The highest cognitive benefits were observed when screen recreation time was converted to NSLT_LA. This study explores various ways in which screen time can be converted. Firstly, simply adjusting the amount of screen time can have cognitive benefits, such as converting SET to SLT. Secondly, the greatest cognitive benefits were seen when SET was converted to NSLT. It is important to note that children's learning and cognitive development are closely intertwined. Interestingly, this study also found that NSLT_LA has a stronger correlation with cognitive ability than NSLT_LWP. Generally speaking, children in early childhood have weak autonomy and require parents to accompany them in learning and provide timely control and guidance (Wang, 2024). Therefore, education experts often recommend that parents spend more time with their children at an early age, particularly in early reading. Theoretically, NSLT_LWP should have significant cognitive benefits. However, the present study does not support this view. There are three possible reasons for this: (1) Through interviews, it was found that kindergartens place great emphasis on children's independent learning after class, and advocate for parents to allow children to explore independently in related learning activities after leaving the kindergarten without seeking help. Therefore, most parents believe that while accompanying learning is important, cultivating their children's independent learning habits is more important. (2) Independence is a crucial factor in early childhood learning and has a significant impact on cognitive development (Peng, 2020), this viewpoint is supported by the fact that the cognitive benefits of converting other periods into NSLT_LA are relatively highest in this study. (3) Research has shown that children often lack quality parental accompaniment, and low-quality parental accompaniment, such as a lack of verbal activities, has been linked to an increase in screen time (Supanitayanon et al., 2020). Finally, converting SET to either STH or ETOK also showed cognitive benefits. Interestingly, the cognitive benefit of converting to ETOK was slightly higher than that of STH, which aligns with current published guidelines.

Third, the association between screen time and cognitive ability varies by discipline and gender. Screen time was more strongly associated with math ability than language ability. Although previous studies have supported the negative associations of language ability and math ability with children's screen time, the relationship between screen time and cognition in early childhood has been controversial (Hu et al., 2020; Supanitayanon et al., 2020). Individual characteristics, parental behavior, and situational factors may influence the association, especially in early childhood when brain networks are rapidly developing (Kostyrka-Allchorne et al., 2017; Hutton et al., 2020). The impact of screen time on cognition in early childhood has been a topic of debate. Some studies suggest that passive screen time, such as watching television or playing video games that do not involve problem-solving or physical activity, may have a negative effect on math achievement and executive functioning in Chinese preschoolers. On the other hand, active screen time, such as playing educational video games or engaging in physical activities, may have a positive impact on their receptive language skills (Hu et al., 2020). The fact that the present study did not examine active screen time may have contributed to this finding. In addition, the variability in the associations between language and math skills and cognitive abilities in the present study may be due to the research instrument, as language and math skills tests have more instruments with different reliability and validity in early childhood. Therefore, although the present study revealed a stronger association between children's screen time and math ability, the present study still suggests that the association between screen time and language ability will show more positive associations due to the differences in the instruments used to measure them. In fact, this study also found some evidence to support the reverse correlation between screen time and children's language ability. For example, an increase in screen time is negatively correlated with boys' language ability, but not for girls. This gender difference may be because girls have a greater advantage in the left brain compared to boys, which is responsible for speech (Han, 2004).

There are some limitations to this paper. First, this study is based on a single sample of children in a large city, and the findings cannot be generalized to the whole country. It can only be said that it has some reference significance in understanding the association between screen time and cognitive development among preschoolers in large cities, and it still needs to be verified in multiple fields based on more sample data in the future. Second, this study is a cross-sectional study rather than a longitudinal analysis, and the association between screen time and cognitive development may show different associations with age and changes in schooling. Finally, the data were collected during the epidemic, and this particular period may have a certain impact on the results of the study, future scholars need to pay attention to this issue.

## 5 Conclusion

This study examined the association between screen time and cognitive ability in 3–6-year-old children from the perspective of balancing time use. The research results not only reinforced existing research findings that the association between screen time and cognitive ability is closely related to the use of screen time, but also further indicated that the negative impact of SET_TV on children's cognition is relatively higher than SET_OED, and there are cognitive benefits when SET is replaced with SLT, NSLT, STH, and ETOK, with the highest benefits observed when SET is substituted with NSLT_LA. Future scholars should explore the direction of adjusting children's screen time from a holistic perspective, not only focusing on the internal structural adjustments of screen time, such as adjusting part of screen entertainment time to screen learning time but also focusing on time outside of screen time, such as sleep time and exercise time.

## Data availability statement

The raw data supporting the conclusions of this article will be made available by the authors, without undue reservation.

## Ethics statement

This study has received ethical approval from the Human Subject Protection Committee of East China Normal University, with approval number HR342-2024. The studies were conducted in accordance with the local legislation and institutional requirements. Written informed consent for participation in this study was provided by the participants' legal guardians/next of kin. Written informed consent was obtained from the individual(s), and minor(s)' legal guardian/next of kin, for the publication of any potentially identifiable images or data included in this article.

## Author contributions

CZ: Writing – original draft, Funding acquisition. ZA: Writing – review & editing, Resources, Investigation. WL: Writing – review & editing, Validation.
