# Supplementary material for: An isochronic substitution benefit study of the effects of screen time on the cognitive abilities of 3–6 children
Source: Front Psychol. 2024 Jul 24;15:1421341. doi: 10.3389/fpsyg.2024.1421341 (PMC11303332; doi:10.3389/fpsyg.2024.1421341)
Supplement: Supplementary file 1 [file Data_Sheet_1.docx]

*Supplementary Table 1 Changes in boys' cognitive abilities after screen time and related factor time were substituted for each other for 10 min/d*

|  | SLT_TV | | SLT_OED | | NSLT_LA | | NSLT_LWP | | STH | | ETOK | | |
| --- | --- | --- | --- | --- | --- | --- | --- | --- | --- | --- | --- | --- | --- |
|  | B  (95% CI) | P | B  (95% CI) | P | B  (95% CI) | P | B  (95% CI) | P | B  (95% CI) | P | B  (95% CI) | P | |
| △ChZ | | | | | | | | | | | | | |
| Model 1 (replaces SET_TV) | **1.03**  (0.13,1.93) | 0.025 | 0.55  (-0.39,1.49) | 0.252 | 0.61  (-0.03,1.24) | 0.060 | 0.20  (-0.57,0.97) | 0.608 | **0.51**  (0.04,0.97) | 0.033 | 0.13  (-0.40,0.65) | 0.634 | |
| Model 2 (alternative to SET_OED) | 0.76  (-0.46,1.98) | 0.222 | 0.28  (-1.08,1.64) | 0.689 | 0.33  (-0.63,1.30) | 0.497 | -0.07  (-1.21,1.07) | 0.904 | 0.24  (-0.63,1.10) | 0.592 | -0.14  (-1.00,0.71) | 0.740 | |
| Model 3 (replaces SLT_TV) | -- | -- | -0.48  (-1.82,0.85) | 0.477 | -0.42  (-1.39,0.54) | 0.385 | -0.83  (-1.97,0.31) | 0.154 | -0.52  (-1.38,0.33) | 0.229 | **-0.90**  (-1.80,-0.005) | 0.049 | |
| Model 4 (replaces SLT_OED) | 0.48  (-0.85,1.82) | 0.477 | -- | -- | 0.06  (-0.98,1.10) | 0.912 | -0.35  (-1.53,0.84) | 0.566 | -0.04  (-0.96,0.88) | 0.932 | -0.42  (-1.35,0.51) | 0.374 | |
| △MaZ | | | | | | | | | | | | |  |
| Model 1 (replaces SET_TV) | **0.96**  (0.08,1.84) | 0.032 | **1.23**  (0.31,2.14) | 0.009 | **0.86**  (0.24,1.48) | 0.006 | 0.32  (-0.43,1.07) | 0.402 | **0.61**  (0.15,1.07) | 0.009 | **0.70**  (0.18,1.21) | 0.008 | |
| Model 2 (alternative to SET_OED) | 0.57  (-0.62,1.77) | 0.346 | 0.84  (-0.49,2.16) | 0.216 | 0.47  (-0.48,1.42) | 0.330 | -0.07  (-1.19,1.05) | 0.902 | 0.22  (-0.63,1.07) | 0.610 | 0.31  (-0.53,1.14) | 0.468 | |
| Model 3 (replaces SLT_TV) | -- | -- | 0.26  (-1.04,1.57) | 0.690 | -0.10  (-1.04,0.84) | 0.830 | -0.64  (-1.76,0.47) | 0.259 | -0.35  (-1.19,0.48) | 0.407 | -0.26  (-1.14,0.61) | 0.553 | |
| Model 4 (replaces SLT_OED) | -0.26  (-1.57,1.04) | 0.690 | -- | -- | -0.37  (-1.38,0.65) | 0.477 | -0.91  (-2.06,0.25) | 0.125 | -0.62  (-1.51,0.28) | 0.176 | -0.53  (-1.44,0.38) | 0.252 | |

Note: Row variables are substituted variables and column variables are substitutes. Bold represents significant changes after substitution. SET_TV is screen entertainment time, which includes watching cartoons and other entertainment programs on TV, SET_OED is screen entertainment time which includes using other electronic devices besides TV for entertainment, SLT_TV is screen learning time which includes watching educational programs on TV, SLT_OED is screen learning time which includes using other electronic devices besides TV for learning, NSLT_LA is learning alone without the use of electronic devices, NSLT_LWP is learning with parents without the use of electronic devices, STH is sleep time at home, ETOK is exercise time outside of kindergarten, ChZ stands for Language ability, MaZ stands for Math ability.

*Supplementary Table 2 Changes in girls' cognitive abilities after screen time and related factor time were substituted for each other for 10 min/d*

|  | SLT_TV | | SLT_OED | | NSLT_LA | | NSLT_LWP | | STH | | ETOK | |
| --- | --- | --- | --- | --- | --- | --- | --- | --- | --- | --- | --- | --- |
|  | B  (95% CI) | P | B  (95% CI) | P | B  (95% CI) | P | B  (95% CI) | P | B  (95% CI) | P | B  (95% CI) | P |
| △ChZ | | | | | | | | | | | | |
| Model 1 (replaces SET_TV) | -0.09  (-1.30,1.12) | 0.882 | 0.44  (-0.64,1.53) | 0.422 | 0.26  (-0.57,1.09) | 0.535 | -0.31  (-1.46,0.84) | 0.597 | -0.15  (-0.80,0.51) | 0.664 | 0.32  (-0.36,1.01) | 0.349 |
| Model 2 (alternative to SET_OED) | -0.13  (-1.55,1.30) | 0.860 | 0.41  (-1.30,2.12) | 0.639 | 0.23  (-1.05,1.50) | 0.727 | -0.35  (-1.89,1.20) | 0.660 | -0.18  (-1.35,0.99) | 0.762 | 0.29  (-0.85,1.42) | 0.617 |
| Model 3 (replaces SLT_TV) | -- | -- | 0.54  (-1.01,2.08) | 0.494 | 0.35  (-0.73,1.44) | 0.521 | -0.22  (-1.60,1.17) | 0.756 | -0.05  (-1.02,0.91) | 0.913 | 0.42  (-0.63,1.46) | 0.434 |
| Model 4 (replaces SLT_OED) | -0.54  (-2.08,1.01) | 0.494 | -- | -- | -0.18  (-1.43,1.06) | 0.774 | -0.75  (-2.25,0.74) | 0.322 | -0.59  (-1.65,0.47) | 0.274 | -0.12  (-1.20,0.96) | 0.827 |
| △MaZ | | | | | | | | | | | | |
| Model 1 (replaces SET_TV) | 0.08  (-0.94,1.09) | 0.881 | 0.46  (-0.45,1.37) | 0.323 | **0.75**  (0.05,1.44) | 0.035 | -0.53  (-1.50,0.43) | 0.277 | 0.09  (-0.46,0.64) | 0.739 | 0.40  (-0.17,0.97) | 0.171 |
| Model 2 (alternative to SET_OED) | -0.38  (-1.57,0.81) | 0.532 | 0.002  (-1.43,1.43) | 0.998 | 0.29  (-0.78,1.36) | 0.591 | -0.99  (-2.29,0.30) | 0.133 | -0.36  (-1.35,0.62) | 0.467 | -0.06  (-1.01,0.89) | 0.903 |
| Model 3 (replaces SLT_TV) | -- | -- | 0.38  (-0.91,1.67) | 0.562 | 0.67  (-0.24,1.58) | 0.147 | -0.61  (-1.77,0.55) | 0.301 | 0.02  (-0.79,0.83) | 0.969 | 0.32  (-0.55,1.20) | 0.472 |
| Model 4 (replaces SLT_OED) | -0.38  (-1.67,0.91) | 0.562 | -- | -- | 0.29  (-0.75,1.33) | 0.585 | -0.99  (-2.25,0.26) | 0.120 | -0.37  (-1.25,0.52) | 0.418 | -0.06  (-0.96,0.84) | 0.895 |

Note: Row variables are substituted variables and column variables are substitutes. Bold represents significant changes after substitution. SET_TV is screen entertainment time, which includes watching cartoons and other entertainment programs on TV, SET_OED is screen entertainment time which includes using other electronic devices besides TV for entertainment, SLT_TV is screen learning time which includes watching educational programs on TV, SLT_OED is screen learning time which includes using other electronic devices besides TV for learning, NSLT_LA is learning alone without the use of electronic devices, NSLT_LWP is learning with parents without the use of electronic devices, STH is sleep time at home, ETOK is exercise time outside of kindergarten, ChZ stands for Language ability, MaZ stands for Math ability.
